# Effect of Lithium-Slag in the Performance of Slag Cement Mortar Based on Least-Squares Support Vector Machine Prediction

**DOI:** 10.3390/ma12101652

**Published:** 2019-05-21

**Authors:** Jianghu Lu, Zhexuan Yu, Yuanzhe Zhu, Shaowen Huang, Qi Luo, Siyu Zhang

**Affiliations:** 1 School of Materials Science and Engineering, Nanchang University, Nanchang 330031, China; 5710116133@email.ncu.edu.cn (J.L.); 5701117008@email.ncu.edu.cn (Y.Z.); huangsw@163.com (S.H.); 2 School of Qianhu, Nanchang University, Nanchang 330031, China; yuzhexuan@email.ncu.edu.cn; 3 School of Civil Engineering, Guangdong Province Key Laboratory of Durability for Marine Civil Engineering, Shenzhen University, Shenzhen 518060, China

**Keywords:** least-squares support vector machine, lithium-slag, cement, compressive strength, anti-chloride ion permeability, fluidity

## Abstract

There is a universally accepted view that environmental pollution should be controlled while improving cement mortar natural abilities. The purpose of this study is to develop a green cement mortar that has better compressive strength and anti-chloride ion permeability. Two industrial wastes, lithium-slag and slag, were added to cement mortar, and the role of lithium-slag was to activate slag. In addition, to save economic and time costs, this paper also used the least-squares support vector machine (LS-SVM) method to predict the property changes of cementitious-based materials. Then multiple natural abilities of samples, including compressive strength, anti-chloride ion permeability, and fluidity, were tested. In addition, LS-SVM and traditional support vector machine (SVM) were used to train and forecast the performance, including compressive strength. The results show that lithium-slag can activate slag to improve the compressive strength, anti-chloride ion permeability of mortar, and LS-SVM sharpens accuracy by 11% compared to SVM.

## 1. Introduction

Cement mortar has a wide range of applications, and reinforcement for concrete structures is an important one [1]. The objective of this research is to develop a green cement mortar with better compressive strength and anti-chloride ion permeability. These are important performance parameters in the structural design of cementitious-based materials. Concrete reinforced by this proposed can improve the corrosion resistance and compressive strength of buildings. In recent years, studies have found that adding proper industrial waste to cement can sharpen the performance. Cement mortar containing silica fume can improve the compressive strength of cement, discovered by Zelić et al. [2] Tafraoui et al. [3] ascertained the good durability properties of Ultra-High-Performance Concrete (UHPC) containing metakaolin. Chi et al. [4] studied the effects of fly ash/slag ratio and liquid/binder ratio on the strength of alkali-activated fly ash/slag (AAFS) mortar. Luo et al. [5] established that lithium-slag can improve the compressive strength of alkali-activated slag concrete. However, many researchers only researched its compressive strength. This paper studies not only compressive strength, but also fluidity and anti-chloride ion permeability of cement containing lithium-slag and slag. In addition, if using conventional methods, such as property tests, there are disadvantages in the process that are long, costly, complicated, and highly variable for results. This paper used better methods to predict the properties.

Machine learning has been widely used to evaluate and predict the performance of cement in recent years [6,7,8,9,10,11,12]. Demir [6] showed that a fuzzy logic algorithm can estimate the elastic modulus from compressive strength of concrete. Goktepe et al. [7] proved that the neural network model can predict the sulfate expansion of various cements containing natural pozzolan and fly ash. In this study, the least-squares support vector machine (LS-SVM) was used to predict the performance of lithium-slag and slag cement, including compressive strength, anti-chloride ion permeability, and fluidity. LS-SVM [13] transformed the inequality constraint into an equality, which simplified the process. It improves speed and has better generalization ability concurrently.

In China, the Xinjiang Lithium Salt Factory emits 64,000 to 80,000 tons of lithium-slag per year. Lithium-slag is the tail residue left after lithium mica is extracted from lithium. After the extraction of potassium, lithium, lanthanum, cerium, and other metals, 90% of the lithium-slag still remains in the lithium mica concentrate. There is 300,000 tons slag in the annual output of 20,000 tons of the lithium carbonate production line. According to incomplete statistics, the amount of lithium-slag discharged from China is more than 800,000 tons yearly [14,15,16]. If it cannot be reused effectively, it will occupy a lot of space and pollute the environment. Therefore, recycling lithium-slag as cement mortar admixture is beneficial to both environment and economy.

Based on the above, lithium-slag was incorporated into slag cement to develop an environmentally friendly cement mortar with better compressive strength and anti-chloride ion permeability. Through experiments, the performance of cement mortar with different proportions of lithium-slag and slag was measured, including compressive strength, anti-chloride ion permeability, and fluidity. After this, models were built with the support vector machine (SVM) and LS-SVM methods. The models were then used to predict the corresponding properties of different ratios of mortar and to obtain predicted values. Combining the above three performances predicted by the model with the actual production demand, it is possible to obtain a practical lithium-slag ratio, which saves time and economic costs. Finally, we compared the predicted value with the measured one, and the performances of SVM and LS-SVM were contrasted by indicators such as the coefficient of determination.

## 2. Experiment

### 2.1. Material Introduction

The cement was 42.5 grade Portland and produced by Jiangxi Yadong Cement Co Ltd, Jiujiang, China. Its apparent density was 3.1 $g/{\mathrm{cm}}^{3}$. The water-reducing agent comes from Jiangxi Dite Company, Nanchang, China. Its property is shown in Table 1. The sand was produced in Jiujiang, Jiangxi. It has a silica content of more than 96%, a loss on ignition of less than 0.4%, and a mud content of less than 0.2%. The lithium-slag was extracted from lithium mica provided by Jiangxi Yufeng Lithium Industry Co Ltd, Ganzhou, China and slag from Xinyu Iron and Steel Plant, Xinyu, China. The specific chemical composition of slag, lithium-slag, and cement are shown in Table 2 and the specific particle size distribution are shown in Table 3.

### 2.2. Performance Test

There are many factors that affect the performance, such as the geometry of the sample, the curing method, the proportion of the admixture, and the amount of the admixture. Lithium-slag was first separately mixed into the cement to test compressive strength and fluidity of the cement. The water, sand and water-reducing agents were 210 g, 1350 g, and 2 g, respectively. The initial mass of cement was 450 g. After that, we substituted lithium-slag for slag, and water-reducing agent was used to control the volume of water. Each cement sample contained 2 g of water-reducing agent and 1350 g of sand. The curing temperature was 20±2 °C and humidity above 90%. The ratio of ingredients is shown in Table 4.

#### 2.2.1. Compressive Strength

The 40 mm × 40 mm × 160 mm prism samples were casted for the cement mortar sample according to “Determinations for isotopes of lead, strontium, and neodymium in rock samples (Chinese Standard GB/T 17671-1999)” [17]. After the samples were cured and demolded, one set of samples was added to water for 7 days, and another for 28 days. Then, the compressive strength of cement was tested using the “YAW4206 microcomputer controlled automatic pressure testing machine” manufactured by SANS. The loading rate was (2400±200) N/s. Compressive strength $R_{c}$ calculated in Newtons per square millimeter (MPa) as follows:

(1) $$R_{c}=\frac{F_{c}}{A}$$

where $F_{c}$ is the maximum load at break (N), *A* is the area of the compressed part (mm${}^{2}$) (40 mm × 40 mm = 1600 mm${}^{2}$).

#### 2.2.2. Anti-Chloride Ion Permeability

Cylindrical samples with diameter of 100±1 mm and a height of 50±2 mm were prepared according to the “Standard for Test Methods of Long-Term Performance and Durability of Ordinary Concrete (Chinese Standard GB/T 50082-2009)” [18]. After being cured and demolded, the samples were added to water for 56 days. The anti-chloride ion permeability was tested using the electric flux method. After vacuum saturation, the samples were placed in the standard test environment. The electrical flux values were automatically recorded using “PER-6A chloride ion permeator” manufactured by Beijing Shourui Co. Ltd, Beijing, China.

#### 2.2.3. Fluidity

Each group of cement mortar samples were prepared in accordance to the “Test method for fluidity of cement mortar (Chinese Standard GB/T 2419-2005)” [19]. Subsequently, the fluidity was tested using “NLD-3 cement mortar fluidity meter”. The samples were lifted vertically and jolted 25 times. The test results took the arithmetic mean of the diameters in two perpendicular directions to the nearest 1 mm.

## 3. Methodology

The SVM [20] is a concrete realization of the principle of dimensionality and its optimization goal is to minimize the risk of structuring. It seeks the best compromise between model complexity and learning ability based on finite sample information, making itself a powerful non-linear estimation tool. The SVM is suitable for solving the practical problems of a small amount of data, non-linearity, overfitting, large number of input parameters, and local minimum points.

The LS-SVM further enhances these advantages of SVM. It uses the least-squares linear system as a loss function, replacing the traditional quadratic programming method of SVM [21]. In SVM, least-squares can convert inequality constraints into equality constraints, simplifying the solution of Lagrangian multiplier alpha. The quadratic programming problem is transformed into a solution to linear equations. Consequently, the solution speed and convergence accuracy of the problem are improved.

Let us assume that the training set ${\left(x_{i},y_{i}\right)}_{N}$, where *i* varies from 1 to *N*, $x_{i}$ is the input vector, $y_{i}$ is the output vector, and *N* is the number of samples. In a regression problem, the goal is to find a hyperplane function. The linear function is expressed as follows [22]:

(2) y(x)=ω·φ(x)+b

where: ω is a normal vector; *b* is the intercept; ϕ(x) is a non-linear mapping from the input space to the output space. The next step is to minimize the Euclidean norm, which is $\frac{1}{2}\left\|\omega \right\|$. Then we introduce error variables *e* with regularization constant γ bigger than 0. γ acts as a noise term to avoid overfitting. So it can be written like this:

$$\mathrm{Minimize}\;\frac{1}{2}{∥\omega ∥}^{2}+\frac{1}{2}\gamma \sum_{k=1}^{N}e_{k}^{2}$$

(3) $$s.t.\left\{\begin{array}{c} y_{k}=\omega \cdot \varphi \left(x_{k}\right)+b+e_{k}\\ k=1,2,\ldots ,N \end{array}\right.$$

We use the duality theory and introduce a Lagrangian operator to obtain the following formula [23].

(4) $$\begin{array}{cc} L= & \frac{1}{2}{∥\omega ∥}^{2}+\frac{1}{2}\gamma \sum_{k=1}^{N}e_{k}^{2}-\sum_{k=1}^{N}\alpha_{k}\left[\omega \cdot \varphi \left(x_{k}\right)+b+e_{k}-y_{k}\right] \end{array}$$

The conditions for optimality are given by:

(5) $$\left\{\begin{array}{l} \frac{\partial L}{\partial \omega }=0\to \omega =\sum_{k=1}^{N}\alpha_{k}\varphi \left(x_{k}\right)\\ \frac{\partial L}{\partial b}=0\to \sum_{k=1}^{N}\alpha_{k}=0\\ \frac{\partial L}{\partial e_{k}}=0\to \alpha_{k}=\gamma e_{k},\left(k=1,\ldots ,N\right)\\ \frac{\partial L}{\partial \alpha_{k}}=0\to \omega \cdot \varphi \left(x_{k}\right)+b+e_{k}-y_{k}=0 \end{array}\right.$$

We define the kernel function $K\left(x_{i},x_{j}\right)=\varphi \left(x_{i}\right)\varphi \left(x_{j}\right)$ as a symmetric function that satisfies Equation (5). Then we convert the optimization problem to solve linear equations, as follows:

(6) $$\begin{array}{c} \left[\begin{array}{cccc} 0 & 1 & \ldots & 1\\ 1 & K(x_{1},x_{1})+2/c & \ldots & K(x_{1},x_{N})\\ \vdots & \vdots & \ldots & \vdots \\ 1 & K(x_{N},x_{1}) & \ldots & K(x_{N},x_{N})+2/c \end{array}\right]\cdot \left[\begin{array}{c} b\\ \alpha_{1}\\ \vdots \\ \alpha_{N} \end{array}\right]=\left[\begin{array}{c} 0\\ y_{1}\\ \vdots \\ y_{N} \end{array}\right] \end{array}$$

Finally, we use the LS-SVM to find α and *b* and obtain a non-linear prediction model:

(7) $$f\left(x\right)=\sum_{k=1}^{N}\alpha_{k}K\left(x_{i},x_{j}\right)+b$$

The three common kernel functions are as follows:

1. Linear kernel function
  (8) $$K\left(x_{i},y_{i}\right)=x_{i}y_{i}$$
2. Polynomial kernel function
  (9) $$K\left(x_{i},y_{i}\right)={\left(x_{i}y_{i}+c\right)}^{d}$$
3. Radial basis function (RBF) kernel function
  (10) $$K\left(x_{i},x_{j}\right)=\exp\left(-\gamma {∥x_{i}-x_{j}∥}^{2}\right)$$

## 4. Model Development

In this study, there were models for predicting three properties, including compressive strength, fluidity, and electric flux. The input parameter *X* was the amount of cement, slag, lithium-slag, and water in the cement mortar, and the output parameter *y* was the compressive strength, fluidity, and electric flux.

The train-and-test technique [24] was used to develop the model, which is one of the most common ways to establish a learning algorithm for a given data set.

Combined with the actual situation of the data set, the data was divided into a training set and a test set following the ratio of 2:1. We used common statistical methods to evaluate the performance of the models, for instance: correlation coefficient (CC), coefficient of determination ($R^{2}$), root mean square error (RMSE), mean absolute error (MAE), and mean absolute percentage error (MAPE) [25]. Their formulae are as follows:

(11) $$CC=\frac{N\sum_{i=1}^{N}y_{i}f_{i}}{\sqrt{N\sum {\left(y_{i}\right)}^{2}-{\left(\sum y_{i}\right)}^{2}}\sqrt{N\sum {\left(f_{i}\right)}^{2}-{\left(\sum f_{i}\right)}^{2}}}$$

(12) $$R^{2}=1-\sum_{i=1}^{N}{\left(y_{i}-f_{i}\right)}^{2}/\sum_{i=1}^{N}\left(y_{i}-\bar{y}\right)$$

(13) $$RMSE=\sqrt{\frac{1}{N}\sum_{i=1}^{N}{\left(y_{i}-f_{i}\right)}^{2}}$$

(14) $$MAE=\frac{1}{N}\sum_{i=1}^{N}\left|y_{i}-f_{i}\right|$$

(15) $$MAPE=\frac{1}{N}\sum_{i=1}^{N}\left|\frac{y_{i}-f_{i}}{y_{i}}\right|\times 100\%$$

where *N* is the total number of the data samples, $y_{i}$ represents each measurement data, $f_{i}$ is each prediction data, and $\bar{y}$ represents the average of all measurement data.

The closer $R^{2}$ and CC are to 1, the more accurate and reliable the model is [9].

We perform normalization before training to eliminate the influence of different dimensions of the input parameters on the prediction results performed. We set the normalization interval to [0, 1]. The calculation formula is as follows:

(16) $$X_{norm}=2\times \frac{X-X_{min}}{X_{max}-X_{min}}+1$$

where $X_{min}$, $X_{max}$ and $X_{norm}$ denote the minimum, maximum, and scaled value of the *X* data sample, respectively [26].

**Figure 1:**
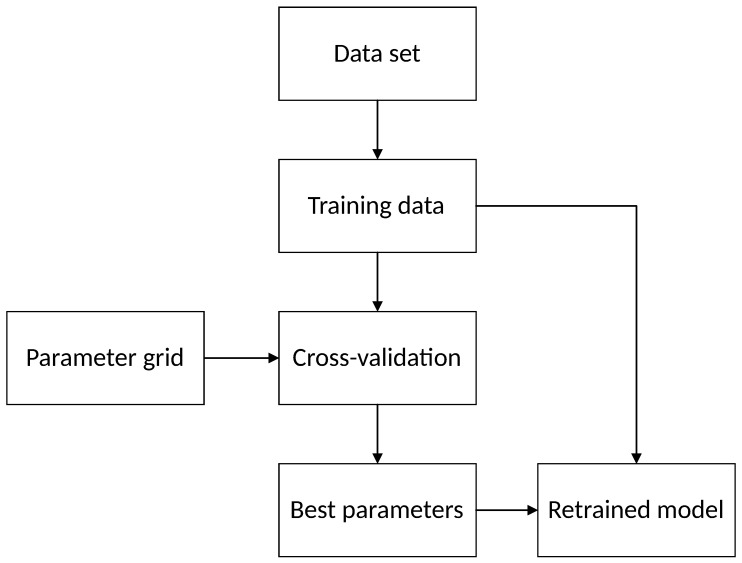
Steps to the Gridsearch method.

The SVM used in this study was implemented based on the Python3 sklearn library [27]. The parameters of the model were determined by the Gridsearch (GS) method. The training data in the data set was trained with the parameters in the parameter grid, and the cross-validation obtains the best parameters. Finally, retrained the model based on the best parameters. The specific flow is shown in Figure 1.

The modeling process includes the following steps:

1. Convert the data of each parameter into a dimensionless one in the range of [0, 1] through the normalization formula.
2. Divide the 33 samples into a training set and a test set in a 2:1 ratio randomly.
3. Use the GS to determine the optimal parameters of the three models. The parameters were the same for the SVM and LS-SVM. In addition, results are shown in Table 5.
4. Compare the results predicted by the three models. The outcome is shown in Table 6.

## 5. Results and Discussion

**Figure 2:**
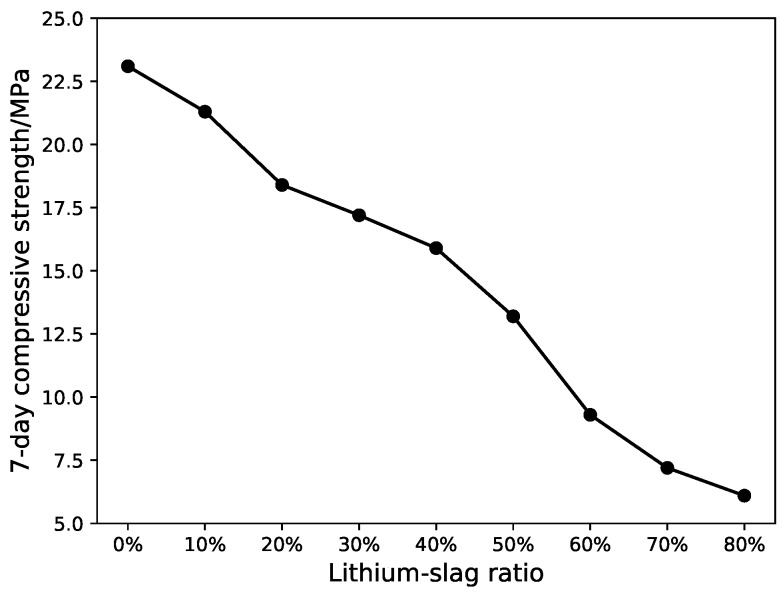
7-day compressive strength of lithium-slag cement mortar.

**Figure 3:**
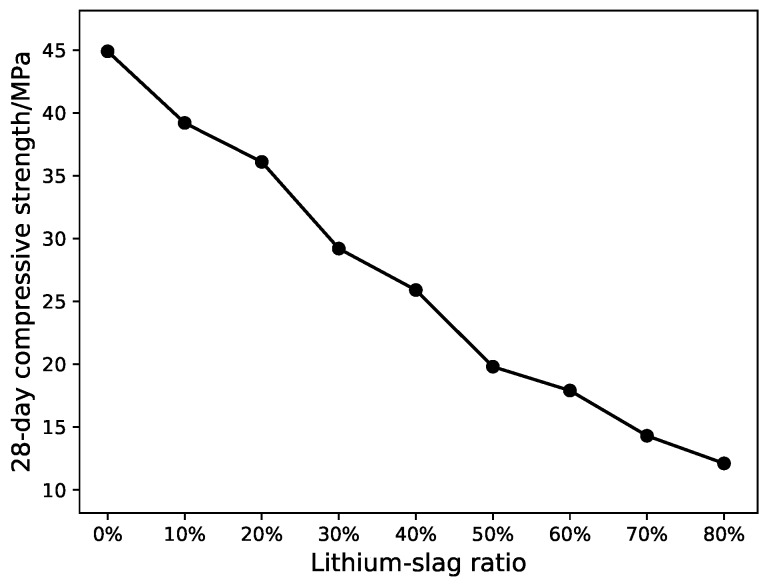
28-day compressive strength of lithium-slag cement mortar.

**Figure 4:**
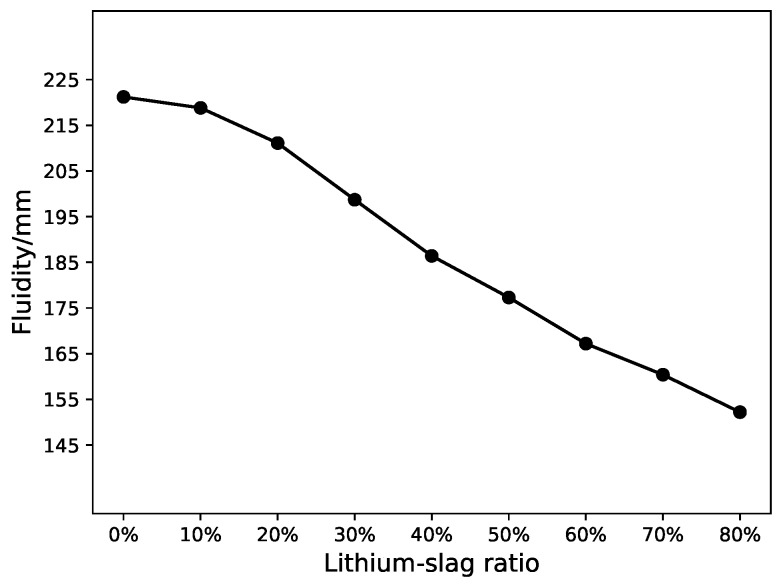
Fluidity of lithium-slag cement mortar.

From Figure 2, Figure 3 and Figure 4, the compressive strength and fluidity of the cement will fall as the proportion of lithium-slag raises, when the lithium-slag was directly mixed with cement. The strength of cement mainly depends on the hydration reaction of mineral C3S and C3A in the cement clinker. Due to the incorporation of lithium-slag, the content of clinker minerals in the grit reduces, resulting in a decrease in strength. At the same time, the lithium-slag structure is loose and has many internal pores, so the water absorption is large. When the amount of lithium-slag in the cement goes up, the water demand will swell, and the fluidity will decrease.

**Figure 5:**
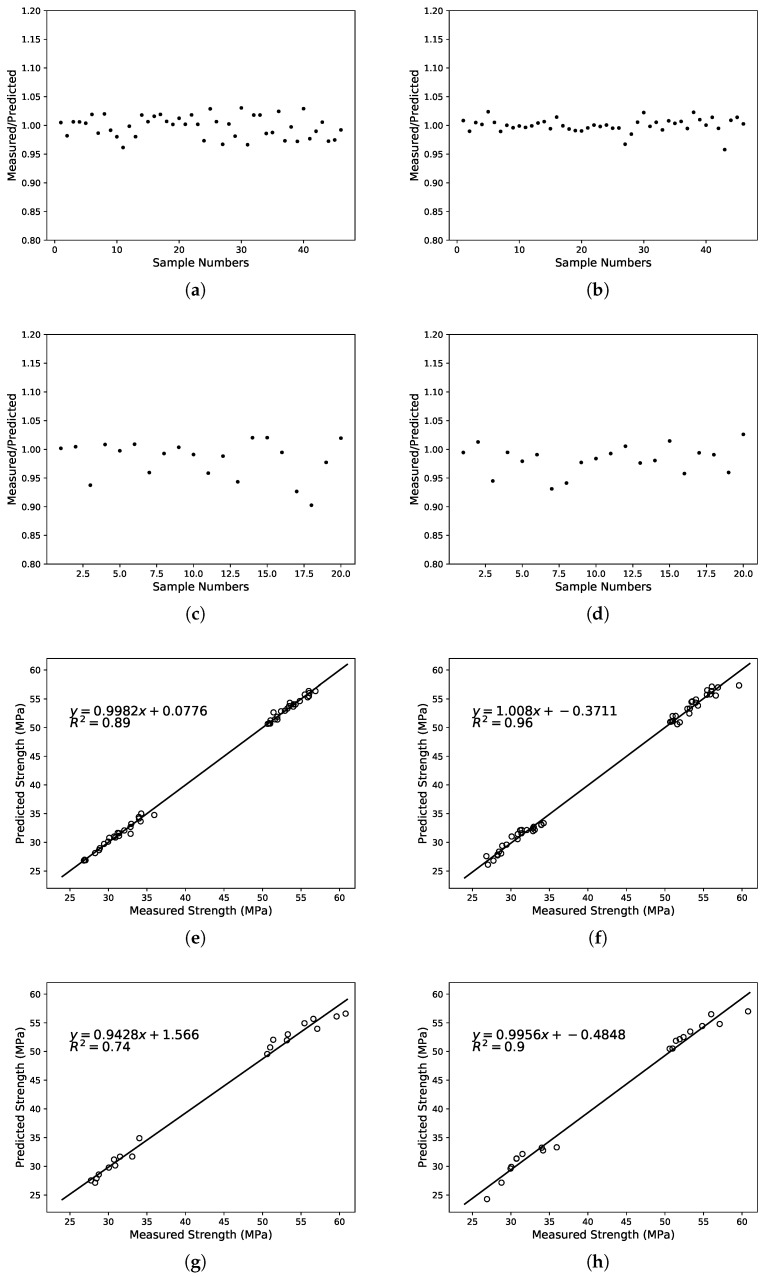
Comparison of the ration between the measured and predicted compressive strength using SVM and LS-SVM. The ratio: (**a**) SVM in training set, (**b**) LS-SVM in training set, (**c**) SVM in test set, (**d**) LS-SVM in test set; The correlation: (**e**) SVM in training set, (**f**) LS-SVM in training set, (**g**) SVM in test set, (**h**) LS-SVM in test set.

**Figure 6:**
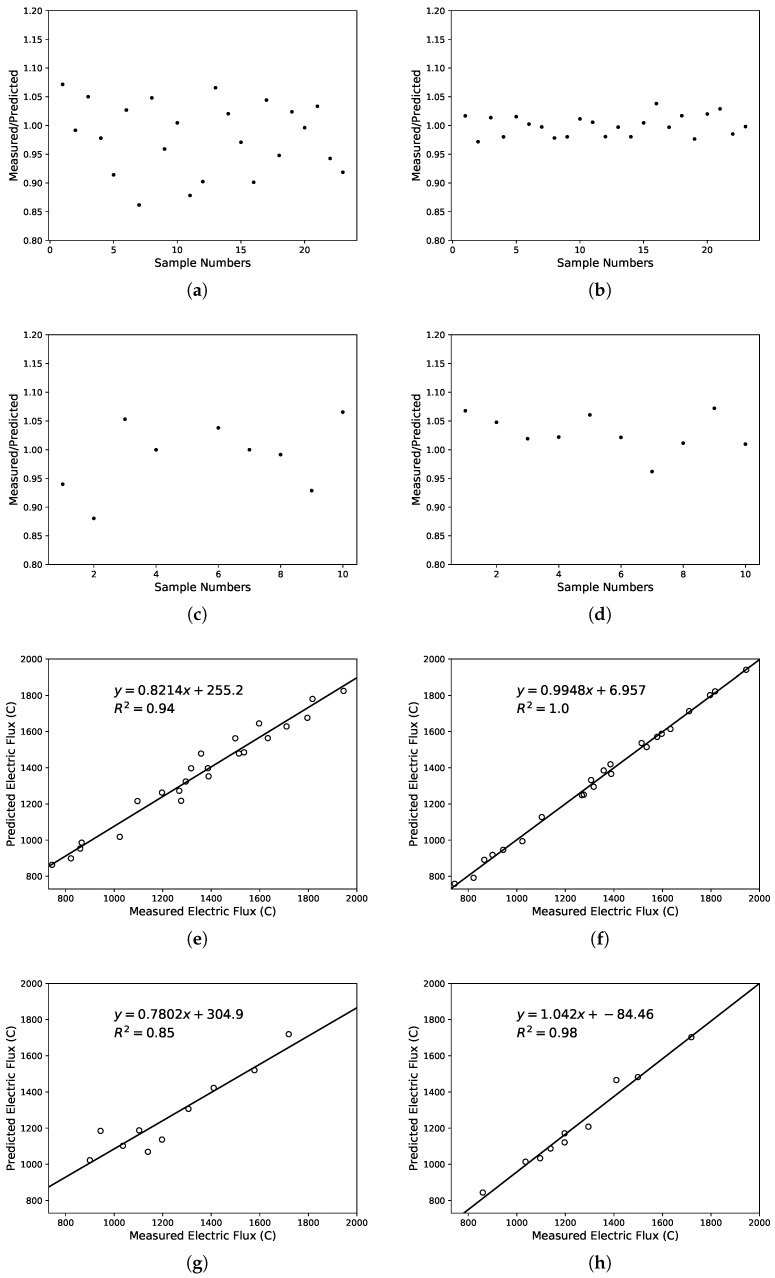
Comparison of the ration between the measured and predicted electric flux using SVM and LS-SVM. The ratio: (**a**) SVM in training set, (**b**) LS-SVM in training set, (**c**) SVM in test set, (**d**) LS-SVM in test set; The correlation: (**e**) SVM in training set, (**f**) LS-SVM in training set, (**g**) SVM in test set, (**h**) LS-SVM in test set.

**Figure 7:**
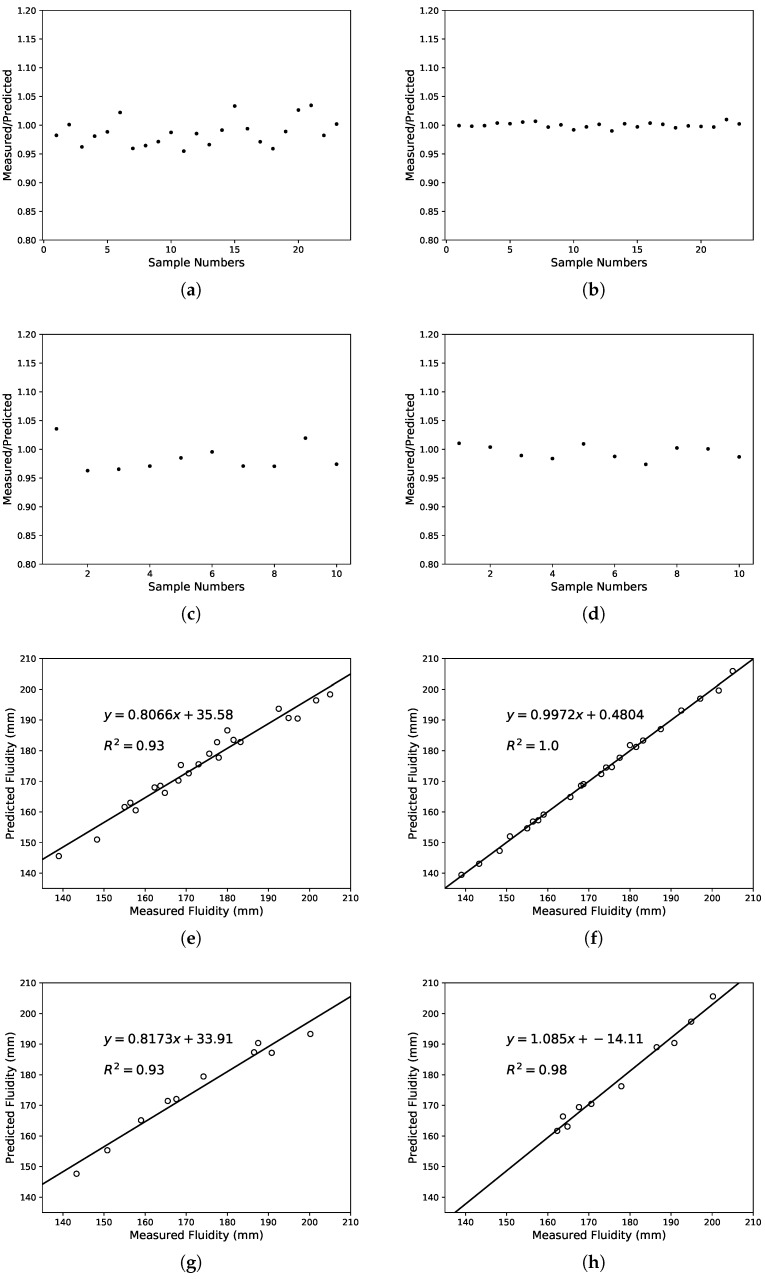
Comparison of the ration between the measured and predicted fluidity using SVM and LS-SVM. The ratio: (**a**) SVM in training set, (**b**) LS-SVM in training set, (**c**) SVM in test set, (**d**) LS-SVM in test set; The correlation: (**e**) SVM in training set, (**f**) LS-SVM in training set, (**g**) SVM in test set, (**h**) LS-SVM in test set.

In this study, performance prediction was done using SVM and LS-SVM. From Figure 5, Figure 6 and Figure 7, the predicted values of the three performance indicators were compared with measured values. The results of the SVM are displayed on the left and the LS-SVM on the right. The scatter plot shows the predicted/measured value ratio and presents error measurements with $R^{2}$ coefficient. Table 7 shows the error measurements obtained by the models, including training set and test set.

An attempt was made to show the influence of lithium-slag and slag on compressive strength using the SVM and LS-SVM. The experimental results are presented in Table 6. The results showed that lithium-slag and slag influenced the compressive strengths. It can be seen from this table that as the proportion of lithium-slag instead of slag increases, the compressive strength would grow first, and then decrease. The filling value and chemical composition of the lithium-slag were maximized first, and the cement strength reached peak. After that, the incorporation of lithium-slag lowered the amount of cementitious material, and the excessive lithium-slag particles debased the gelation and the compressive strength of the samples.

Another goal was made to show the influence of lithium-slag and slag on fluidity and chloride ion permeation resistance using the SVM and LS-SVM. Experimental results are shown in Table 8. From the results, it can be found that lithium-slag and slag reduce the fluidity of cement and boost the anti-chloride ion permeability. The permeability was related to the compactness and void structure of cement. The incorporation of lithium-slag reduces the irregular capillary channels, which were connected to each other due to the formation of water. Simultaneously, the replacement of cement by lithium-slag shriveled the hydration speed in the early gelling system, making the structure development relatively perfect. In addition, the CSH gel produced by the secondary hydration of the lithium-slag made the internal structure of the concrete more compact and greatly enhanced the resistance to chloride ion penetration. Moreover, as the amount of lithium-slag continued to climb, the viscosity of the cement rose and the fluidity diminished, due to the large volume of water required for the lithium-slag itself.

Furthermore, the performance of the lithium-slag and slag cement mortar was predicted using the SVM and LS-SVM. It can be seen from Table 7 that the $R^{2}$ and CC values of the SVM and LS-SVM both were very close to 1. According to the scatter plot, it can be discovered that the variation between the measured and predicted values of LS-SVM was less than that of SVM in any case. The linear regression lines show that the performance predicted by these three models was basically in line with expectations. After comparing $R^{2}$, it can be seen that LS-SVM performed much better than SVM. The performance of the two methods was calculated using the common benchmark. As can be seen from Table 6 and Table 8, it can be determined that the model constructed by SVM and LS-SVM both can be used to assess the performance of the lithium-slag and slag cement mortar. In addition, the model built with LS-SVM was more accurate and robust.

In contrast, Zelić et al. [2] established that silica fume can improve the compressive strength of cement. This study found that the mixture of lithium-slag and slag not only has the same function, but also boosts the anti-chloride ion permeability of cement. The latter can enhance more performance and thus has a wider application scenario, even if the effect of increasing the compressive strength is not as obvious as the former. In addition, this research uses a more scientific and effective method, LS-SVM, to help the actual production. In general, LS-SVM accepts local optimal solutions, and the accuracy should be lower than SVM. However, both of them fit the data well in performance predictions in this study. Furthermore, LS-SVM has stronger generalization ability, and shows better performance for the dataset in this research.

## 6. Conclusions

This study aims to develop a green cement mortar with better performance, and forecast its changes with a better machine-learning method. In the present research, most studies have only studied the compressive strength properties of related cements. This paper not only studies the compressive strength of lithium-slag and slag cement, but also the fluidity and anti-chloride ion permeability, and used SVM and LS-SVM to predict these properties. We used common statistical methods to evaluate the performance of the models and compared the traditional SVM and LS-SVM.

The following conclusions could be drawn from this investigation:

1. Lithium-slag can activate slag and improve the compressive strength and anti-chloride ion permeability of cement. However, excessive lithium-slag will attenuate its fluidity and compressive strength. Therefore, this cement mortar can be used in special scenes with corrosion resistance and high compressive strength requirements, such as chemical plant floor, and chemical laboratory floor.
2. The LS-SVM can predict not just the compressive strength of this cement mortar, but the electric flux and fluidity. Comparing the three models comprehensively, it can be extracted that the LS-SVM used in this paper has an 11% improvement in accuracy compared to the previous SVM.
3. The study confirms the possibility of recycling lithium-slag and slag in cement production. There is a new way to reduce the accumulation of lithium-slag, which scales back environmental pollution.

Combined with pre-designed ratio information, the performance of cement mortar can be predicted according to the model. Due to several factors affecting actual production, such as raw material quality and curing environment, engineers need to combine their practical experience to predict the performance of mortar based on the proportion of raw materials. For future work, cement mortar can be further blended with different types of industrial waste, to reduce the environmental pollution of industrial waste.

References

## Figures and Tables

**Table 1:** The property of water-reducing agent.

Property	Value
Density (g/mL)	1.07±0.02
Solid content (%)	20±2
pH	6∼8
Chloride ion content (%)	≤0.02
Alkali content (%)	≤0.2

**Table 2:** The chemical composition of slag, lithium-slag and cement (%).

Oxide	SiO_2_	Al_2_O_3_	CaO	Fe_2_O_3_	MgO	SO_3_	Na_2_O	K_2_O	TiO_2_
Lithium-slag	47.62	21.56	2.02	0.48	0.12	0.03	10.68	3.05	3.46
Slag	33.72	14.02	41.53	0.33	7.17	1.88	0.31	0.43	0.4
Cement	22.61	5.67	60.12	3.35	1.57	2.27	-	-	-

**Table 3:** The particle size distribution of slag, lithium-slag, and cement (%).

Material	0∼1 μm	1∼5 μm	5∼10 μm	10∼20 μm	20∼40 μm	40∼85 μm	85∼ μm
Lithium-slag	5.6	34.9	15.3	20.4	19.0	4.8	0
Slag	9.4	25.1	14.3	24.9	22.7	3.6	0
Cement	7.02	19.33	18.17	22.76	24.86	5.85	0

**Table 4:** Details of mix proportions for lithium-slag and slag cement mortar.

Data No.	Water/g	Cement/g	Slag/g	Lithium-Slag/g	Lithium-Slag/%
1	210	225	225.0	0.0	0
2	210	225	211.5	13.5	3
3	210	225	198.0	27.0	6
4	210	225	184.5	40.5	9
5	210	225	171.0	54.0	12
6	210	225	157.5	67.5	15
7	210	225	144.0	81.0	18
8	210	225	130.5	94.5	21
9	210	225	117.0	108.0	24
10	210	225	103.5	121.5	27
11	210	225	90.0	135.0	30
12	210	270	180.0	0.0	0
13	210	270	166.5	13.5	3
14	210	270	153.0	27.0	6
15	210	270	139.5	40.5	9
16	210	270	126.0	54.0	12
17	210	270	112.5	67.5	15
18	210	270	99.0	81.0	18
19	210	270	85.5	94.5	21
20	210	270	72.0	108.0	24
21	210	270	58.5	121.5	27
22	210	270	45.0	135.0	30
23	200	315	135.0	0.0	0
24	200	315	121.5	13.5	3
25	200	315	108.0	27.0	6
26	200	315	94.5	40.5	9
27	200	315	81.0	54.0	12
28	200	315	67.5	67.5	15
29	200	315	54.0	81.0	18
30	200	315	40.5	94.5	21
31	200	315	27.0	108.0	24
32	200	315	13.5	121.5	27
33	200	315	0.0	135.0	30

**Table 5:** Selected parameters.

Method	Kernel	Gamma	C
SVM	RBF	1	1000
LS-SVM	RBF	1	1000

**Table 6:** Comparison of compressive strength measured and predicted results.

Data No.	Experimental Results/MPa		SVMRresults/MPa		LS-SVM Results/MPa	
	7 days	28 days	7 days	28 days	7 days	28 days
1	26.90	50.87	24.28	51.06	26.97	50.77
2	28.78	51.03	27.15	51.13	28.65	51.24
3	29.96	51.39	29.60	52.03	30.12	52.05
4	30.72	52.91	31.32	53.28	31.17	52.94
5	31.20	53.99	32.11	54.34	31.64	53.65
6	32.88	57.11	31.96	54.79	31.49	53.96
7	30.10	53.45	31.02	54.47	30.79	53.73
8	29.43	53.28	29.60	53.47	29.73	52.99
9	28.74	51.91	28.07	52.15	28.56	51.88
10	27.73	50.68	26.82	50.99	27.53	50.64
11	27.02	50.61	26.11	50.48	26.88	49.56
12	28.27	51.93	27.87	50.91	28.13	51.40
13	30.05	52.41	29.89	52.50	29.77	52.85
14	30.74	53.55	31.36	54.57	31.01	54.32
15	31.50	56.03	32.14	56.38	31.67	55.49
16	33.09	59.62	32.18	57.32	31.69	56.11
17	31.38	56.09	31.58	57.10	31.13	56.03
18	30.87	55.87	30.56	55.79	30.14	55.28
19	28.88	54.29	29.40	53.83	28.98	54.05
20	28.46	51.43	28.38	51.86	27.90	52.65
21	28.27	51.60	27.74	50.58	27.13	51.42
22	26.82	50.99	27.59	50.52	26.83	50.69
23	30.89	54.03	31.45	54.89	30.84	54.04
24	32.06	55.45	32.14	55.72	32.08	54.93
25	32.96	55.49	32.70	56.50	33.22	55.76
26	33.96	56.87	33.10	56.98	34.15	56.36
27	35.95	60.79	33.31	56.99	34.77	56.60
28	34.25	56.00	33.34	56.50	35.02	56.37
29	34.01	56.60	33.24	55.58	34.90	55.69
30	33.94	54.84	33.03	54.44	34.42	54.62
31	34.18	53.23	32.76	53.32	33.66	53.32
32	32.87	53.17	32.46	52.45	32.69	51.95
33	31.39	50.99	32.16	52.01	31.61	50.72

**Table 7:** Error measurements for predicting performance based on the SVM and LS-SVM.

Performance	Method	Training Set					Test Set				
		CC	$R^{2}$	RMSE	MAE	MAPE	CC	$R^{2}$	RMSE	MAE	MAPE
Compressive Strength	SVM	0.946	0.89	0.755	0.635	1.6	0.916	0.739	1.43	1.0	2.59
	LS-SVM	0.978	0.956	0.459	0.333	0.833	0.948	0.899	1.41	1.09	2.35
Electric Flux	SVM	0.983	0.943	78.2	68.1	5.63	0.939	0.852	98.2	71.5	6.89
	LS-SVM	0.998	0.996	20.4	18.1	1.53	0.989	0.977	50.0	43.0	3.55
Fluidity	SVM	0.983	0.925	4.58	4.01	2.34	0.984	0.926	4.79	4.49	2.65
	LS-SVM	0.999	0.998	0.786	0.615	0.356	0.992	0.984	2.41	1.94	1.07

**Table 8:** Comparison of electric flux and fluidity measured and predicted results.

Data No.	Experimental Results		SVM Results		LS-SVM Results	
	Electric Flux/C	Fluidity/mm	Electric Flux/C	Fluidity/mm	Electric Flux/C	Fluidity/mm
1	1578	205.0	1520	198.4	1571	205.9
2	1514	201.6	1479	196.4	1537	199.6
3	1410	192.5	1422	193.7	1466	193.1
4	1389	187.5	1353	190.3	1366	187.0
5	1268	180.0	1273	186.6	1249	181.8
6	1103	177.5	1188	182.8	1128	177.7
7	1036	175.6	1102	179.0	1014	174.6
8	900	173.0	1022	175.6	918	172.4
9	860	170.6	953	172.6	844	170.5
10	822	168.1	899	170.2	792	168.6
11	744	163.7	863	168.5	759	166.4
12	1796	200.2	1676	193.3	1801	205.6
13	1710	194.9	1628	190.7	1713	197.3
14	1633	186.5	1564	187.3	1614	189.0
15	1535	181.5	1485	183.5	1514	181.2
16	1386	174.2	1398	179.4	1419	174.5
17	1306	168.7	1306	175.3	1332	169.1
18	1276	165.5	1217	171.4	1251	164.9
19	1197	162.3	1137	168.0	1171	161.7
20	1139	159.0	1069	165.1	1087	159.1
21	1023	156.4	1018	163.0	994	156.9
22	866	155.0	986	161.6	891	154.6
23	1945	197.1	1825	190.5	1940	197.0
24	1817	190.8	1780	187.2	1822	190.4
25	1719	183.2	1719	182.8	1703	183.3
26	1597	177.9	1645	177.7	1588	176.2
27	1499	167.6	1563	172.1	1482	169.4
28	1358	164.8	1478	166.2	1385	163.1
29	1317	157.7	1397	160.5	1295	157.3
30	1295	150.8	1324	155.3	1208	152.0
31	1197	148.3	1263	151.0	1121	147.3
32	1096	143.3	1216	147.7	1033	143.1
33	944	139.0	1185	145.6	946	139.4

## References

[1] Afridi M., Ohama Y., Demura K., Iqbal M. (2003). Development of polymer films by the coalescence of polymer particles in powdered and aqueous polymer-modified mortars. Cement Concr. Res..

[2] Zelić J., Krstulović R., Tkalčec E., Krolo P. (2000). The properties of Portland cement-limestone-silica fume mortars. Cement Concr. Res..

[3] Tafraoui A., Escadeillas G., Vidal T. (2016). Durability of the ultra high performances concrete containing metakaolin. Constr. Build. Mater..

[4] Chi M.C., Liu Y.C. (2013). Effects of Fly Ash/Slag Ratio and Liquid/Binder Ratio on Strength of Alkali-Activated Fly Ash/Slag Mortars. Appl. Mech. Mater..

[5] Qi L., Shaowen H., Yuxuan Z., Jinyang L., Weiliang P., Yufeng W. (2017). Influence of lithium slag from lepidolite on the durability of concrete. IOP Conf. Ser. Earth Environ. Sci..

[6] Demir F. (2005). A new way of prediction elastic modulus of normal and high strength concrete—Fuzzy logic. Cement Concr. Res..

[7] Goktepe A., Inan G., Ramyar K., Sezer A. (2006). Estimation of sulfate expansion level of PC mortar using statistical and neural approaches. Constr. Build. Mater..

[8] Chou J.S., Pham A.D. (2013). Enhanced artificial intelligence for ensemble approach to predicting high performance concrete compressive strength. Constr. Build. Mater..

[9] Ashrafian A., Amiri M.J.T., Rezaie-Balf M., Ozbakkaloglu T., Lotfi-Omran O. (2018). Prediction of compressive strength and ultrasonic pulse velocity of fiber reinforced concrete incorporating nano silica using heuristic regression methods. Constr. Build. Mater..

[10] Chou J.S., Tsai C.F., Pham A.D., Lu Y.H. (2014). Machine learning in concrete strength simulations: Multi-nation data analytics. Constr. Build. Mater..

[11] Yu Y., Li W., Li J., Nguyen T.N. (2018). A novel optimised self-learning method for compressive strength prediction of high performance concrete. Constr. Build. Mater..

[12] Sarıdemir M. (2011). Empirical modeling of splitting tensile strength from cylinder compressive strength of concrete by genetic programming. Expert Syst. Appl..

[13] Suykens J.A., Lukas L., Vandewalle J. Sparse approximation using least squares support vector machines. Proceedings of the 2000 IEEE International Symposium on Circuits and Systems. Emerging Technologies for the 21st Century (IEEE Cat No. 00CH36353).

[14] Zeng C. (2012). Study on Preparing Lightweight Ceramsite Using Leaching Residual Slag of Lepidolite Ore. Master’s Thesis.

[15] Qin Y., Li X., Guo Y. (2016). Orthogonal experimental research on mix ratio design of C30 recycled concrete with lithium slag. Concrete.

[16] Zhai M., Zhao J., Wang D. (2018). Applying Lithium Slag Powders to Cement-based Materials as Supplementary Cementitious Component: An Overview. Mater. Rep..

[17] (1999). GB/T 17671-1999: Method of Testing Cements–Determination of Strength.

[18] (2009). GB/T 50082-2009: Standard for Test Methods of Long-Term Performance and Durability of Ordinary Concrete.

[19] (2005). GB/T 2419-2005: Test Method for Fluidity of Cement Mortar.

[20] Smola A.J., Schölkopf B. (2004). A tutorial on support vector regression. Stat. Comput..

[21] Wang H., Hu D. Comparison of SVM and LS-SVM for regression. Proceedings of the 2005 International Conference on Neural Networks and Brain.

[22] Cortes C., Vapnik V. (1995). Support-vector networks. Mach. Learn..

[23] Weixing Q., Fei Y., Yizhou L., Jiaxing X., Guowen H., Bangxiong Z. (2017). Prediction of Compressive Strength of Super Early Strength Concrete Based on Support Vector Machine. Subgrade Eng..

[24] Ahmed T., Burley E., Rigden S., Abu-Tair A.I. (2003). The effect of alkali reactivity on the mechanical properties of concrete. Constr. Build. Mater..

[25] Behnood A., Verian K.P., Gharehveran M.M. (2015). Evaluation of the splitting tensile strength in plain and steel fiber-reinforced concrete based on the compressive strength. Constr. Build. Mater..

[26] Özcan F., Atiş C.D., Karahan O., Uncuoğlu E., Tanyildizi H. (2009). Comparison of artificial neural network and fuzzy logic models for prediction of long-term compressive strength of silica fume concrete. Adv. Eng. Softw..

[27] Pedregosa F., Varoquaux G., Gramfort A., Michel V., Thirion B., Grisel O., Blondel M., Prettenhofer P., Weiss R., Dubourg V. (2011). Scikit-learn: Machine Learning in Python. J. Mach. Learn. Res..

